# Static Balance From the Short Physical Performance Battery Compared With BTrackS Among Older Adults

**DOI:** 10.1093/geroni/igaf122.4013

**Published:** 2025-12-31

**Authors:** Jethro Raphael Suarez, Kworweinski Lafontant, Daniel Goble, Joon-Hyuk Park, Michael Dino, Ladda Thiamwong

**Affiliations:** University of Central Florida, Florida, United States; University of Central Florida, Florida, United States; Oakland University, Rochester, Michigan, United States; Daegu Gyeongbuk Institute of Science and Technology, Daegu, Republic of Korea; University of Central Florida, Florida, United States; University of Central Florida, Florida, United States

## Abstract

The static balance portion of the Short Physical Performance Battery (SPPB) has rarely been compared directly with gold standard force plate balance assessments. This study aimed to compare static balance metrics from a cost-efficient force plate between community-dwelling older adults with low and high SPPB balance scores. The Balance Tracking System (BTrackS), a stationary and simple balance assessment instrument, was used for measuring static balance metrics during a non-visual static balance test. The static balance portion of the SPPB ranges from a total score of 0 to 4 and involves unsupported standing for 10 seconds in a side-by-side, a semi-tandem, and a tandem stance. We cross-sectionally compared center of pressure (COP) path length and sway area from 204 community-dwelling older adults aged 61 to 96 (176 women, mean age = 75 ± 7 years) who completed a BTrackS and SPPB assessment. A total of 146 older adults exhibited a high SPPB balance score (= 4), while 58 exhibited a low SPPB balance score (≤ 3). The Low SPPB Balance group had significantly greater COP path length (p = 0.006) and sway area (p < 0.001) compared to the High SPPB Balance group. These results suggest that the balance score from the SPPB is a valid indicator of static balance performance when compared to a gold standard force plate assessment and therefore, provide evidence for clinicians with limited technological resources to use the SPPB in a similar population and infer postural sway metrics based on high or low SPPB balance scores.

